# Reshaping life after stroke: a grounded theory

**DOI:** 10.1016/j.ijnsa.2025.100348

**Published:** 2025-05-10

**Authors:** Dagmar J.J. van Nimwegen, Sigrid C.J.M. Vervoort, Johanna M.A. Visser-Meily, Lisette Schoonhoven, Janneke M. de Man-van Ginkel

**Affiliations:** a Research Group Proactive Care for Older People Living at Home, Research Center for Healthy and Sustainable Living, HU University of Applied Sciences Utrecht, Utrecht, the Netherlands; b Department of Nursing Science, Julius Center for Health Sciences and Primary Care, University Medical Center Utrecht, Utrecht, the Netherlands; c Department of Rehabilitation, Physical Therapy Science & Sports, UMC Utrecht Brain Center, University Medical Center Utrecht, Utrecht, the Netherlands; d Academic Nursing, Department of Gerontology and Geriatrics, Leiden University Medical Centre, Postzone C-07-Q, P.O. Box 9600, Leiden 2300 the Netherlands

**Keywords:** Stroke, Psychology, Delivery of health care, Psychological well-being, Stroke care pathway, Interprofessional

## Abstract

**Background:**

Stroke patients often experience different consequences, negatively impacting their psychosocial well-being. Since every patient has their individual process and their individual needs, providing personalised stroke care is difficult. Determining what is needed in stroke care is crucial to optimize well-being after stroke.

**Objective:**

To gain understanding of how patients experience the process they go through, which psychosocial needs they experience, and whether the current stroke care matches this process.

**Methods:**

A qualitative study according to the methodology of Grounded Theory, by conducting semi-structured interviews with ten stroke patients who were receiving stroke care in a stroke service.

**Results:**

Patients after stroke go through a basic social process of reshaping life which was characterized by two perspectives – feeling lost and rediscovering yourself. These perspectives could be refined on several themes: focusing on capabilities; setting goals; experiencing emotions; feeling heard or understood; and finding meaning in life. The following themes influence this process: help and understanding from patients’ network; patients’ needs being met in stroke care; and support and motivation from other patients.

**Conclusions:**

Healthcare professionals could use the found process of reshaping life to determine for each patient where they find themselves within this process. They can use this to determine which needs patients experience, and how they can match these needs to support patients in reshaping life to improve patients’ psychosocial well-being after stroke.

**Funding and registration:**

This study was supported by the Taskforce for Applied Research SIA (RAAK.PUB04.010) and was registered at the Dutch Trial Register (NL7440).

**What is already known**

- For many people not only physical but also psychosocial well-being is impacted after stroke

- Every person has their own individual recovery process

- Providing personalized care within stroke care pathways is challenging

What this paper adds

- After stroke people go through a process in which the ultimate challenge is to reshape their life

- The process of reshaping life is characterized by two perspectives: ‘feeling lost’ and ‘rediscovering yourself’

- The process of reshaping life can be influenced by the factors ‘help and understanding from patients’ own network’, ‘patients’ needs being met in stroke care’, and ‘support and motivation from other patients’

Alt-text: Unlabelled box

## 1 Background

For most stroke patients the first year after stroke is characterized by numerous changes in their abilities and daily lives. Stroke patients generally experience many different consequences, such as physical, cognitive, communicative, and psychological consequences (Marsh et al., 2023). These consequences have a major impact on their lives after stroke, which can manifest as lower quality of life (Ramos-Lima et al., 2018), worse relationships (Ramazanu et al., 2019), lower autonomy (Palstam et al., 2019), and less participation in activities, work or education and social relationships (Palstam et al., 2019). Furthermore, the consequences patients experience after stroke often greatly affect their psychosocial well-being (Ferro et al., 2016), where psychosocial well-being can be defined as including good psychological and social well-being, a lack of negative emotions, participation and engagement in activities, social relations, and experiencing self-esteem, self-acceptance, and self-belief (Kirkevold et al., 2018). Low psychosocial well-being is known to lead to worse recovery, higher mortality, and slower rehabilitation (Robinson and Jorge, 2016; Meadmore et al., 2019). In practice, the degree to which stroke consequences influence patients’ psychosocial well-being depends on many factors, among which personality and psychological factors (van Mierlo et al., 2014), social support and other environmental factors (Foley et al., 2019), and emotional adjustment (Smith et al., 2021). Due to the large variety of consequences among stroke patients, attention for psychosocial well-being after stroke is crucial to improve patients’ recovery.

Stroke patients generally experience many different needs, ranging from functional needs to needs in daily lives and psychosocial needs. Whether patients’ needs are being met is partly influenced by the care they receive after stroke (Zawawi et al., 2020), which is generally provided in stroke care pathways. In general, these stroke care pathways comprise several different organizations in which care is provided by interprofessional teams (Clarke and Forster, 2015). Each patient has their own individual route within these pathways, depending on their level of functioning after stroke (Lindblom et al., 2022). Overall, the current stroke care mainly focuses on the first year after stroke since most recovery in activity and participation takes place during this first year (O'Dell et al., 2021; Tse et al., 2019).

Over the last decades, a lot has been invested in both research and healthcare to improve psychosocial well-being after stroke (van Nimwegen et al., 2023). However, in practice most attention seems to be given to functional recovery, and little attention to patients’ psychosocial well-being and needs. Although most nurses and other healthcare professionals involved in stroke care experience psychosocial well-being as important, many differences exist in the psychosocial care they provide and in whether patients’ needs are met by this (Loft et al., 2019). Since psychosocial well-being is influenced by many factors and patients experience many different needs, every patient experiences their own individual process after stroke. As this process is not always parallel to the pathway in which patients receive stroke care, providing personalized care within these pathways is difficult. This leads to unmet patient needs in the currently provided care, which negatively influences patients’ experienced psychosocial well-being (Zawawi et al., 2020). As such, determining what is needed for personalized stroke care is crucial to optimize patients’ psychosocial well-being after stroke. Therefore, the aim of this study is to gain a rich understanding of how patients experience the process they go through after stroke, how this influences their psychosocial well-being, and what (psychosocial) needs they experience during this process. Furthermore, we aim to get insight into whether the current stroke care, provided by nurses and other healthcare professionals, matches the process patients go through and their needs in this process.

## 2 Methods

This study was reported following the COnsolidated criteria for REporting Qualitative research (COREQ) Checklist (Tong et al., 2007).

### 2.1 Study design

To understand and generate a theory of the process stroke patients go through, a qualitative study was performed according to the methodology of Grounded Theory described by Glaser (Glaser, 1978). This method generates a theory that accounts for a pattern, the basic social process, explaining what is going on around a main concern, what themes are present and what factors bring out the change in the process.

Due to the social perspective of this study, we believe there is not one ‘true’ situation. The approach takes into account that although researchers can find an objective view of the situation, this view may be influenced by researchers’ own experiences and expertise. This approach results in a theory which best reflects the data, without claiming this theory to be the one and only truth (Glaser, 1978). To understand the process people go through after stroke, we tried to get as close to the population of stroke patients as possible.

### 2.2 Sampling and recruitment

A purposive sample was used by selecting stroke patients who had experienced an ischemic, hemorrhagic, or subarachnoid hemorrhagic stroke up to one year ago and were receiving treatment for rehabilitation after stroke. Patients were eligible to participate when they were 18 years or older. Patients were not eligible when they did not master the Dutch language or experienced communicative or cognitive difficulties to such an extent that participation in an interview was too burdensome or impossible.

This study was part of a larger research project – UPACT-project: Psychosocial Care After Stroke – in which the researchers have collaborations with five stroke services, which include one or more hospitals, geriatric and specialist rehabilitation organizations and home care organizations. The division between geriatric rehabilitation and specialist rehabilitation is specific for the Dutch stroke care system. Patients capable of more intense rehabilitation are admitted to specialist rehabilitation, while more severely affected patients receive care in geriatric rehabilitation. All organizations belonging to the five stroke services were approached for participation in a previous study of our UPACT-project. Within this previous study, in each organization, a contact person – usually a nurse or another healthcare professional – asked eligible patients for permission to be approached for participation. Patients were approached to reach maximum variation based on demographic characteristics, stroke characteristics, and (type of) organization in which they received care. The researchers (DN, SdV) asked for written informed consent based on verbal and written information. In case of communicative difficulties, a partner or family member was involved in the informed consent process. Additionally, patients were asked the following characteristics: i) setting where the patient received stroke care; ii) demographic characteristics: sex, age, living situation, level of education; and iii) stroke-related factors: time since stroke, type of stroke*.*

For the current study, patients were sampled from a group of patients who participated in the previous study. All participants of this previous study met our purposive sampling characteristics. The researchers (DN, SdV) approached 25 patients who participated in the previous study of our UPACT-project for participation in this study, based on the organization in which they received stroke care. We strived for one participant in each of the 15 participating organizations to achieve maximum variation in (type of) organizations. In total, 10 people agreed to participation in this study, after which an interview was scheduled. The other 15 people did not participate due to the following reasons: not wanting to participate, being unable to participate – either physically or emotionally – or being discharged from the organization before participation was possible. If, during the informed consent procedure, it was noticed that communicative or cognitive difficulties made it impossible to have a conversation, this patient was excluded from participation in an interview. This led to the exclusion of one patient. All patients were aware that the interviewers were researchers and were not involved in the care process.

### 2.3 Data collection

Ten semi-structured interviews were conducted face-to-face in a quiet room of the participating organization or at the patient’s home to ensure the sole presence of the patient and researcher. A topic list was constructed based on literature, in consultation with stroke patients who did not participate in this study, and discussions within the research team – consisting of a PhD student (DN), a senior researcher in nursing science, with elaborate experience in stroke care and psychosocial well-being after stroke (JMG), a senior researcher in nursing science, with elaborate experience in qualitative research (SV), a professor in nursing science (LS), and a rehabilitation physician and professor of rehabilitation medicine (JVM). The topic list was tested on content and clarity in a pilot interview with one stroke patient who did not participate in the study and adapted accordingly. The following topics were explored: patients’ experiences with stroke care; patients’ psychosocial well-being after stroke; and healthcare professionals’ attention for psychosocial well-being in stroke care. All interviews started with the same question as icebreaker: “I understand you have had a stroke. Can you tell me something more about that?” Probing questions were used if needed to address all topic, such as: It is quite normal for stroke patients to often have all kinds of feelings, these can be both positive and negative. Do you ever discuss your feelings with others? To what extent does the healthcare provider pay attention to your feelings? Can you tell something about what this was like for you?

Interviews were conducted between April 2019 and February 2020 by one trained qualitative researcher (DN) and a research assistant (SdV). One researcher (DN) worked as a PhD student with expertise in nursing science and conducting qualitative research and as a teacher at a bachelor’s degree in nursing. This interviewer had a great affinity for patient care and interest in neurology. The research assistant (SdV) worked as a teacher at the bachelor’s degree in social work. This interviewer had a didactic background and was very interested in conducting research. The first interviewer (DN) conducted five interviews alone. To align the manner of interviewing, both interviewers (DN, SdV) conducted three interviews together, where one of them conducted the interview while the other listened or the other way around. These interviews were discussed elaborately after each interview. Lastly, the second interviewer (SdV) conducted two interviews alone. Interviewers were not known to the patients before recruitment in the previous study of our UPACT-project. Interviews were audio recorded and lasted between 33 and 81 min, with an average of 52 min. Data collection and data analysis were conducted iteratively; initial theoretical ideas formed during data analysis were used to further elaborate on topics and refine the content of the topic list for the following interviews, as is appropriate according to theoretical sampling (Glaser, 1978).

### 2.4 Data analysis

Interviews were transcribed verbatim and analyzed by coding them conform the methodology of Grounded Theory (Glaser, 1978), supported by Atlas.ti version 8.4.20.0 (Scientific Software Development GmbH, Berlin).

The interviews were analyzed using an iterative, inductive process to identify the main concern and the basic social process that explains what is going on in the stroke patients. (Glaser, 1978). The first three interviews were analyzed using open coding by three researchers (DN, JMG, SV) independently, after which their interpretations were discussed until consensus was reached. The next two interviews were analyzed using open coding by the first author and discussed with the research team. Next, the first author performed axial coding on the first five interviews to construct initial categories and themes by refining the open codes and relating them to each other. To ensure validity, axial analysis was randomly performed on about 50% of these five interviews by the other two researchers independently. Axial analyses were discussed within the research team until consensus was reached. Next, selective coding was performed in these five interviews to construct the theory that accounts for the main concern, the consequent basic social process, and the related themes forming the pattern and the variation in those themes. The researchers used constant comparison during joint meetings by going back to previous interviews after each step of analysis to check their interpretations and answer remaining questions in all interviews. The constructed theoretical concept was compared for similarities and differences with the previously analyzed interviews as well as the remaining five interviews to confirm or refute the theoretical concept based on all previously analyzed and new data. The theoretical concept was adapted accordingly, after which researchers again checked all ten interviews to confirm or refute this. This was repeated until data saturation of concepts was reached based on all ten interviews, resulting in the theory. To check understandability and appropriateness, the theory was discussed with three stroke patients who had not been previously involved in this study in any way and were adjusted accordingly. Next, the theory was translated into English by the first author and discussed elaborately with the research team until consensus about translation was reached. The translated theory was checked among fifteen experts in nursing science and rehabilitation care and were finalized accordingly.

Patients’ characteristics about setting, sex, living situation, level of education, type of stroke and time since stroke were analyzed using descriptive statistics.

### 2.5 Ethics

The Medical Research Ethics Committee (MREC) of the University Medical Centre Utrecht exempted this study from the Medical Research Involving Human Subjects Act (WMO)-test (18/759). The study was registered at the Dutch Trial Register (NL7440).

### 2.6 Credibility

In qualitative research credibility is of major importance to ensure the study truly reflects the experiences of the studied population. Credibility can be influenced by the researchers’ sensitivity, their self-awareness, their training, and the use of consistent methodology (Glaser, 1978). The research team consisted of a PhD student (DN), a research assistant (SdV), two senior researchers in nursing science (JMG, SV), a professor in nursing science (LS), and a rehabilitation physician and professor of rehabilitation medicine (JVM). Most researchers were experts in the field of nursing care, of which two were experts in the field of rehabilitation after stroke. The first interviewer (DN) was trained in qualitative interviewing, which was confirmed by the research team by elaborately discussing the first conducted interview. To train the second interviewer (SdV), three interviews were conducted in which both interviewers were present, after which these were discussed elaborately. The interviewers (DN, SdV) both did not work in the field of stroke care, which enabled them to objectively conduct the interviews. Due to a great interest in patient care and neurology, the interviewers were able to empathize with the studied population.

Both the role of the interviewers as well as of other researchers in the research team can be of influence on the data collection and analysis. Within all steps of data collection and analysis the personal experiences and expectations or assumptions of all researchers were discussed elaborately. In practice, during each step of data analysis the researchers (DN, SV, JMG) discussed their thoughts about the data and constantly checked if these thoughts were truly grounded within the data and not just based on own experiences or assumptions. Additionally, we are aware of the possibility of psychologically burdened participants, as many stroke patients experience psychosocial problems – e.g. symptoms of depression or anxiety. During our joint meetings we specifically focused on separating the basic social process, and the related themes from any underlying psychological factors. In practice, this meant that in our discussions we critically discussed if the continuum of ‘reshaping life’ would also be correct without any underlying psychological problems. These discussions were partly based on our own knowledge and expertise, but we constantly checked the data. To ensure consistency in methodology, during all steps of data collection and analysis, theoretical memos were written by one researcher (DN) and discussed with the other researchers (SV, JMG). By using this approach of reflexivity, we ensured data collection and analysis were free of assumptions and subjective biases as much as possible.

## 3 Results

Most participants received care in a rehabilitation organization (*n* = 7), had an ischemic stroke (*n* = 7), and had a recent stroke (≤3 months) (*n* = 8). The median age of participants was 75 years (IQR=19). Patients’ characteristics are summarized in Table 1.

**Table 1:** Patient characteristics.

	Total group (*N* = 10)	
	N (%)	Median (IQR)
**Sex**		
Male	5 (50%)	
**Age**		75 (19)
**Living situation**		
Independently alone	4 (40%)	
Independently together	5 (50%)	
Other	1 (10%)	
Educational level*		
Low	2 (20%)	
Middle	7 (70%)	
High	1 (10%)	
**Setting where patient receives care**		
Hospital – inpatient care	1 (10%)	
Hospital – outpatient care	2 (20%)	
Geriatric rehabilitation	5 (50%)	
Medical specialist rehabilitation – inpatient care	2 (20%)	
**Time since stroke**		
1–2 weeks	1 (10%)	
2–3 weeks	1 (10%)	
1–2 months	4 (40%)	
2–3 months	2 (20%)	
6–7 months	1 (10%)	
>12 months	1 (10%)	
**Type of stroke**		
Ischemic	7 (70%)	
Hemorrhagic	2 (20%)	
Subarachnoid hemorrhagic	1 (10%)	

⁎ Educational levels: lowest – primary school; low – lower vocational education and pre-vocational secondary education; middle – secondary vocational education, senior general secondary education and pre-university education; high – higher professional education and university education.

## 4 Reshaping life after disruption due to stroke

After suffering a stroke, the main concern stroke patients experience was disruption of life. The perceived impact of the change of self, the sense of disability and dependence on others after stroke, as well as the uncertainty about recovery and the duration of the recovery trajectory, cause life to be experienced as disrupted. The life disrupted by stroke affects the meaning of life and stroke patients' identities as they have to relate to their life after stroke, which is related to what they nevertheless still are capable of and what not, maybe never again.

*… that I came to die. I had fully anticipated that. Thus. But I get to stay a while longer. That's nice anyway. Yes you've been through so much and that does play a role. … well, your whole doings, because you never forget this. And that would be shameful if you forgot. (R 3)*

*… but because of the brain attacks. And yes, that's a completely different story. And that takes much longer, and that's much more intense … You don't know what's possibly ahead… it's so drastic. (R 1)*

*Because I mean it's already a shock that you get a haemorrhage like that, then you're already thinking ‘oh boy’ what's happening to my body?’, ....En I said it was a.., I was building a block tower, that was my life, and then just something had to happen and then that bottom block was taken out from under it and then that whole block tower was hanging again, yeah, it was falling apart again. (R 4)*

To deal with the main concern of ‘disruption of life’, the basic social process all participants went through after stroke was ‘reshaping life’, meaning that they needed to find a way to reshape their life after stroke. During this process of reshaping life, they reoriented themselves towards the life ahead. This process comprises a continuum in which each participants followed their individual pathway. The speed and direction of this process varied from patient to patient. How patients went through this process was determined by how they emotionally related to the disruption of life due to stroke: feeling lost or rediscovering themselves. This process of reshaping life after stroke is characterized by how individuals act, what they feel and how they give meaning to their situation five themes are differentiated: 1) focusing on capabilities; 2) setting goals; 3) experiencing emotions; 4) feeling heard or understood; and 5) finding meaning in life. Within each theme, participants may find themselves in either a perspective that emphasizes feelings of loss or a perspective emphasizes rediscovering yourself.

Which perspective prevails in the patient's perception can be influenced by help and understanding from patients’ own network; patients’ needs being met in stroke care; and support and motivation from other patients.

**Fig. 1:**
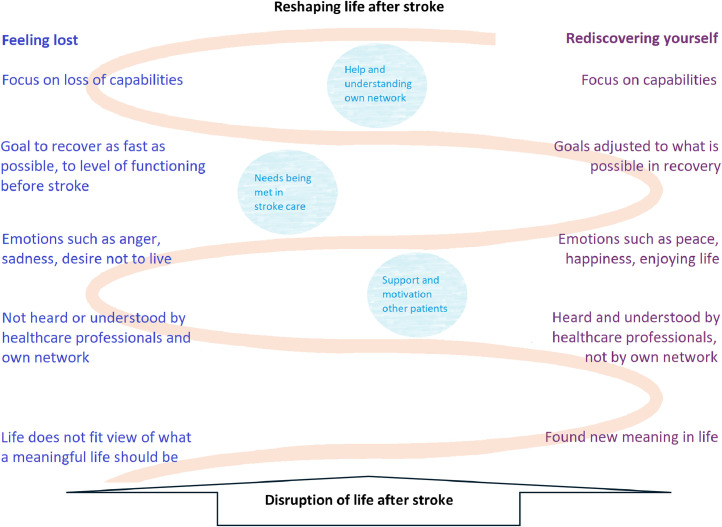
Process of reshaping life after stroke.

This process of reshaping life is depicted in Fig. 1.

### 4.1 Feeling lost

Feeling lost was characterized by patients who mainly focused on their loss of capabilities, expressed as participants only wanting to return to their lives from before stroke. These people hated being dependent on others.

*Everything you have to do, you need someone (. . .) Now you always have to ask everywhere and everyone (. . .) I just hate that. Because I prefer being independent. (R5)*

Furthermore, these participants either had no goals – because they did not want to get disappointed – or their goal was to recover as fast as possible and/or to their level of functioning before stroke– even if it was known this goal probably could not be achieved. This disposition was characterized by mostly experiencing emotions such as anger – with themselves, their healthcare professional, or the situation – sadness, feeling vulnerable, and the desire not to live. Other experienced emotions were fear or anxiety, being in shock, insecurity, feeling powerless, frustration, and feeling tormented.

*(. . .) I couldn’t get up, and I just wanted to die. I have never in my whole life, I have never been depressed and I just wanted to die. (R4)*

Additionally, participants who felt lost, did not feel heard or understood, both by healthcare professionals and by their own partner, family and/or friends. These people had a need to discuss their emotions, but often did not know how to discuss this or with whom.

*A healthcare professional already knows what is coming and already has his answer ready, but I wonder at such moments if he listens when I tell my story. (R2)*

Feeling lost meant that participants experienced their life did not fit their view of what a meaningful life should be, and their view of the future had changed.

*That’s not my idea of life (. . .) Especially before my first stroke, I was living life to the fullest (. . .) Now I’m finally at a point of ‘that is not my life’. (R1)*

### 4.2 Rediscovering yourself

Rediscovering yourself was characterized by patients focusing on their current capabilities, expressed as participants looking for ways to be as independent as possible. These people searched for ways to do as much as possible by themselves and were willing to make adaptations compared to before stroke. For some participants this focus on capabilities came from within themselves, for others this was encouraged by healthcare professionals.

*Stirring up your self-reliance, that’s so important, and they [healthcare professionals] do that on every level (. . .) Whoever you talk to, they awaken that in you. (R4)*

Furthermore, these participants adjusted their goals to what was possible in recovery. They had a good idea about which goals were reachable but left room for refinement when these could not be reached after all. Rediscovering yourself was further characterized by mostly experiencing emotions such as happiness, strength, peace, and a feeling of enjoying and appreciating life.

*You can see nature and the nice weather again, and I can really enjoy that. Lovely. I did that before too, but in a different way (. . .) I appreciate it more, I guess. (R3)*

These participants often felt heard and understood by healthcare professionals. They discussed their emotions with healthcare professionals and experienced this as pleasant and useful. They generally did not discuss their emotions with their own partner, family and/or friends and felt unheard by them.

*If I want to talk about something, I go to the nurses. (. . .) They [family] do not have to know everything (. . .) You always have a sympathetic ear [from nurses], while at home they sometimes say ‘just don’t say anything’. (R3)*

Participants who rediscovered themselves experienced having found a new meaning in life, even though this usually differed from before stroke. These people found a way to live a meaningful life, despite not being able to do everything they could before stroke.

### 4.3 Help and understanding from patients’ own network

Partners, family and friends – hereafter described as ‘network’ – usually had an important influence on participants’ daily lives and emotions and shaped the perspective within basic social process. Their process of reshaping life was generally influenced by the experiences with their own network. Most participants needed their network to help them with things they were not capable of anymore. However, they experienced limitations in the help others could offer them and their network’s help did not always match their needs, resulting in feeling lost. Furthermore, the participants’ network often tried to motivate them. Sometimes it helped them focusing on rediscovering yourself. However, sometimes help was experienced as counterproductive, reinforcing the feeling of being lost. Although participants expressed the need for their network, they often did not feel fully understood or heard by them. Generally, they felt that people could only understand them if they had experienced a stroke themselves. Furthermore, participants did not always share everything with their network, to avoid burdening them.

*When I talk to my wife, she says ‘it will be alright’, but that’s against better judgement, to encourage me. And my son says ‘dad you’re alive, that is most import-, we still got you’. But I’m disabled. (R2)*

### 4.4 Patients’ needs being met in stroke care

For most participants it was important their needs were met by healthcare professionals; when these were met, they generally experienced their received care as positive, otherwise as negative. This experience influenced their perspective in reshaping life. For participants a few needs were most important to be met. Firstly, clarity about the rehabilitation process. It was important for participants to know where they stood. They needed to know what is possible in recovery, what healthcare professionals do within the rehabilitation process, and why healthcare professionals do this.

*Clarity. Al lot of clarity in what we will do, how it will go, and the steps we have to take. You come here [in organization], then draw up balance of what you’ve had, this is what you have to deal with and this is your room [for improvement], period. (R5)*

Secondly, a personal approach in the care provided was found essential Participants wanted the care they received to be adapted to their personal goals and needs, and to match their personal values and daily lives.

*Specifically look at what your goals are in life, where you want to go, and where you come from (. . .) What is important to you, what will we do first, and then work our way up. (R5)*

Thirdly, for participants it was important to make their own decisions about their rehabilitation process and the care they received. If they were unable to make these decisions, they wanted their partner or family to make these. Fourthly, participants experienced a need to recover as fast as possible; in practice this need could generally not be met.

*Í decide. It is about me (. . .) And when I’m not aware I have to decide, then I pass it on to my wife or our daugther, then they will decide for me. But not the nurse. (R1)*

### 4.5 Support and motivation from other patients

How participants experienced their reshaping life process was related to their contact with other patients. Participants generally experienced such contact as positive. It motivated them to see other patients go through the same process and they experienced a feeling of togetherness and having fun with other patients. Furthermore, patients often shared their experiences and emotions with one another, which was usually experienced as valuable.

*There are the other rehabilitation patients (. . you really need eachother. So, that is nice. That there are people who you can talk to (. .) everyone experiences the same process, of discovering the things you cannot do anymore (R4).*

## 5 Discussion

This study resulted in a theory explaining the basic social process patients go through after stroke (Fig. 1). The main concern of stroke patients is that their lives after stroke is disrupted, and they need to reorient to their life ahead. The process of reshaping life addressed this main concern. Two perspectives characterized this process of reshaping life– feeling lost and rediscovering yourself – which could be acknowledged on several themes. Which perspective prevails in the patient's process of reshaping life can be influenced by the following factors: help and understanding from patients’ own network; patients’ needs being met in stroke care; and support and motivation from other patients.

Reshaping life means that patients need to adapt to their new situation after stroke, for which coping is essential. Coping strategies vary among patients, leading to different outcomes on quality of life and well-being (Lo Buono et al., 2017). We found that how the process proceeds is determined by which perception of recovery - feeling lost or rediscovering life – is dominant. These perceptions, which can be seen as opposites, have also been seen in previous research in patients after stroke. Previous research showed that some patients achieve a feeling of life satisfaction after stroke, whereas others do not (van Mierlo et al., 2016). This process is influenced by patients’ personality and psychological factors (van Mierlo et al., 2015). Although we did not specifically study patients’ personality factors from before stroke, our interviews showed the process of reshaping life can be influenced by all aspects of patients’ lives, including which emotions they experience after stroke. In our study patients were included up to one year after stroke. However, previous research suggests the process of reshaping life can continue for at least up to five years after stroke (Body, 2014).

Focusing on loss of capabilities after stroke was found to be related to reshaping life. Previous research shows difficulties with functioning and being dependent on others negatively influences quality of life, participation, and life satisfaction (van Mierlo et al., 2016). Additionally, stroke patients with a tendency to focus on negative aspects generally experience lower health-related quality of life (van Mierlo et al., 2014). This is underlined by a previous study which describes that after stroke people need to adapt and adjust their life, because their capabilities have changed (Matérne et al., 2022). Differences among participants in focus on loss of capabilities or focus on their current capabilities suggests that during the first year after stroke some people may – partially – adapt and adjust their lives, while others may not or to lesser extent.

Our interviews show large differences in goal setting among stroke patients. The importance of goal setting in rehabilitation has been widely studied. This is described as a collaborative process between patients and healthcare professionals (Scobbie et al., 2009). Previous research shows that patients’ personal recovery goals have a major impact on their motivation and their engagement in rehabilitation (Firth et al., 2023). Nevertheless, our interviews show patients’ needs regarding goal setting are often not met in practice, as healthcare professionals have too little attention for patients’ goals. (Firth et al., 2023) showed patients to be dissatisfied with the care they receive when their personal goals and healthcare professionals’ goals do not match (Firth et al., 2023). This suggests collaborative goal-setting between patients and healthcare professionals to be of major importance. (Scobbie et al., 2009) suggest – based on behaviour change theories – that goal setting should include two phases; the first focusing on developing goals, and the second on performing actions to achieve these goals (Scobbie et al., 2009). Our interviews may suggest a lack of these two phases in current stroke care, leading to many differences in the experiences of collaborative goal setting among our participants.

Furthermore, our interviews show that for stroke patients it is important to feel heard and understood; since it is one of the themes within reshaping life. Most participants, however, felt unheard or misunderstood by their own network This can be explained by family members being affected by their loved one's stroke themselves. A previous review shows after stroke stress levels increase in both stroke patients and their spouses, many spouses experience symptoms of depression after the stroke, spouses experience their own coping process, and relationships between patients and spouses often change after stroke (Ramazanu et al., 2019). Because both patients and spouses are affected, many difficulties in communication and conflicting interests may arise. Similarly, relationships with other family members – such as children – are expected to change. (Matérne et al., 2022) found that for stroke patients it is important to feel useful and valuable to other people. Although this study confirms that relationships often change after stroke, these authors found this change can also be positive, resulting in deepened social relationships (Matérne et al., 2022).

Additionally, some participants did not feel heard and understood by healthcare professionals. Feeling heard and understood can be considered a sign that person-centred care is implemented, as person-centred care focuses on individuals’ preferences and autonomy, in contrast to healthcare professionals making the decisions (American Geriatrics Society 2016). Our findings showed patients’ need to make their own decisions is currently sometimes, but not always, met by healthcare professionals. This suggests that person-centred care may currently not be implemented optimally yet.

Our interviews show that whether patients have found a new meaning in life relates to their process of reshaping life. This corresponds to previous research which describes that stroke patients have to find a new balance in their life and that the extent to which patients are able to find new perspectives in life greatly affects their life satisfaction (Body, 2014). Previous research shows that for some people finding meaning in life asks for more changes in their life than for other people after stroke (Matérne et al., 2022).

### 5.1 Methodological considerations and limitations

In this study we followed the methodology of Grounded Theory according to (Glaser, 1978) as much as possible (Glaser, 1978). Based on the elaborate steps of the analysis and the use of constant comparison, data saturation was reached based on our ten interviews. However, because theoretical sampling – in which new participants are approached based on the initial ideas of a theory – was not used, it cannot be determined whether theoretical saturation has been obtained.

An important strength of this study was that part of the interviews were analyzed independently by, and all analyses were discussed among three researchers with varying backgrounds and expertise until consensus was reached. This ensured sensitivity of data-analysis, meaning researchers used their own experiences and expertise to grasp the meaning of data and to subtract concepts which were grounded within these data (Glaser, 1978). Another strength was that non-participating patients were included in developing the topic list, leading to a clear and valid topic list for answering our research question. Furthermore, patients who did not participate in the interviews were included in finalizing the theory that accounts for the main concern, the consequent basic social process, and the related themes forming the pattern and the variation in those themes. As these patients were in a later stage after stroke, this enabled them to reflect more on their entire process after stroke than participating patients, enabling us to check the theory we found based on our interviews.

A limitation was the risk of selection bias. Only patients in need of rehabilitation care after stroke participated in our interviews. As the majority of stroke patients are discharged home after hospitalization, this could lower the generalizability of our results. However, we did include patients who were discharged home in finalizing our analyses. Since these patients recognized themselves in the basic social process of reshaping life and the related themes, we believe the generated theory could mostly apply to these patients as well. Additionally, patients were asked permission by healthcare professionals to be approached for participation. Although the researcher stressed that patients should only be selected based on in- and exclusion criteria, some healthcare professionals tended to ‘protect’ severely affected patients from participation. As such, in some organizations patients who were most severely affected by stroke could have been missed in our study. Consequently, the results of our interviews should be considered carefully for patients with (very) severe consequences after stroke. Furthermore, only patients with no or mild cognitive and/or communicative impairments participated, as patients with more severe consequences were unable to participate in an interview. Additionally, one patient had a stroke more than one year ago, even though we aimed to include patients up to one year after stroke. As we were interested in patients who were receiving stroke care, we believe including this patient did not lead to bias as this patient was receiving care in one of the participating organizations. Lastly, we are aware that some participants may have experienced psychological problems, as these often occur after stroke. We critically discussed the influence of potential psychological problems on the basic social process, and the related themes during our joint meetings, to reduce the risk of bias. However, we cannot conclude with certainty that such problems did not affect our findings as we did not determine if our participants experienced psychological problems. We have consciously chosen not to exclude patients with these problems, as these problems are often prevalent among the population of stroke patients, meaning attention should always be paid to people with such problems.

Although the grounded theory approach can be used to develop a theory, this does not lead to this theory being the only truth perse. Reflections of the truth may be different for different patients and different settings. However, the found theory can be used to give some explanation to the process patients go through after stroke.

### 5.2 Recommendations

The theory found in this study gives explanation to the process of reshaping life patients can experience after stroke. This theory could be used as a guide by nurses and other healthcare professionals for providing care after stroke that matches the needs of individual stroke patients.

Over the past years person-centered care has been receiving increasing attention in healthcare (American Geriatrics Society 2016). Our study raises the question whether the current stroke care is sufficiently person-centered. Being aware of the similarities in needs patients can experience may allow nurses and other healthcare professionals to provide optimal person-centered care after stroke. We recommend healthcare professionals to optimize providing person-centered stroke care by being aware of patients’ needs by: 1) providing stroke patients with as much clarity as possible about what is possible in terms of neurobiological recovery, what patients can expect in the rehabilitation process, what healthcare professionals do and why; 2) involving patients in the decision-making process and letting patients make the final decisions about the care they receive; and 3) adapting the provided care to patient’s personal goals and personal lives. Furthermore, many participants expressed a need to recover as fast as possible, which is often unachievable. The needs described in this study solely focus on the patient’s perspective. However, in providing care the perspective of healthcare professionals is of major importance as well. In daily practice it might be impossible to always meet all individual patient’s needs. Nurses and other healthcare professionals could use described needs to match patients’ perspectives as much as possible. If certain needs cannot be met, healthcare professionals could explain to patients why these needs cannot be met in practice and what can be expected in the rehabilitation process. They could use behavior change theories to help patients in their goal setting process to develop achievable goals. Additionally, healthcare professionals should be aware of the potential influence of patients’ network and could discuss with patients the presence and influence of their partners, family, and friends on their experiences after stroke and patients’ own role in this. Also, they could encourage patients to seek contact with other patients, to support and motivate each other. By using these recommendations, we believe nurses and other healthcare professionals could improve their collaboration with patients in the rehabilitation process after stroke, leading to better outcomes for stroke patients.

In this study we interviewed patients who had a stroke up to one year ago. As reshaping life may continue for much longer (Body, 2014), we recommend to further research this process over longer periods by following the same patients over time and determining their needs at different moments. Further research should also include more patients with (severe) cognitive and/or communicative difficulties, as these patients may experience different needs and such difficulties may influence their pathway in the process of reshaping life. Lastly, in this study mainly patients who received inpatient rehabilitation care were included. As most stroke patients immediately go home after hospital discharge, further research to determine if the process of reshaping life and needs are similar for these patients is recommended.

## 6 Conclusions

This study resulted in a theory that gives explanation to the basic social process patients go through after stroke. The theory found in this study was the basic social process of reshaping life. Two perceptions characterize this process – feeling lost and rediscovering yourself – which can be refined on several themes – focusing on capabilities, setting goals, experiencing emotions, feeling heard and understood, finding meaning in life. Receiving help and understanding from patient’s own network, patients’ needs being met in stroke care, and receiving support and motivation from other patients are related to this process. Nurses and other healthcare professionals could use this theory as a guide for providing care after stroke that matches the needs of individual stroke patients. It is expected this will improve recovery and as such lead to better psychosocial well-being after stroke.

## Registration

This study was registered at the Dutch Trial Register (NL7440).

## Funding sources

**This study was supported by the Taskforce for Applied Research SIA (RAAK.PUB04.010).**

## Data availability statement

All data are available upon request from the corresponding author.

## CRediT authorship contribution statement

**Dagmar J.J. van Nimwegen:** Writing – review & editing, Writing – original draft, Visualization, Validation, Project administration, Methodology, Investigation, Formal analysis, Data curation, Conceptualization. **Sigrid C.J.M. Vervoort:** Writing – review & editing, Writing – original draft, Validation, Methodology, Formal analysis, Conceptualization. **Johanna M.A. Visser-Meily:** Writing – review & editing, Validation, Supervision. **Lisette Schoonhoven:** Writing – review & editing, Validation, Supervision, Project administration. **Janneke M. de Man-van Ginkel:** Writing – review & editing, Writing – original draft, Visualization, Validation, Project administration, Methodology, Funding acquisition, Formal analysis, Conceptualization.

## Declaration of competing interest

The authors declare that they have no known competing financial interests or personal relationships that could have appeared to influence the work reported in this paper.
